# What Actually Confers Adaptive Capacity? Insights from Agro-Climatic Vulnerability of Australian Wheat

**DOI:** 10.1371/journal.pone.0117600

**Published:** 2015-02-10

**Authors:** Brett A. Bryan, Jianjun Huai, Jeff Connor, Lei Gao, Darran King, John Kandulu, Gang Zhao

**Affiliations:** 1 CSIRO Land and Water Flagship and Agriculture Flagship, Waite Campus, Urrbrae, South Australia, Australia; 2 College of Economics and Management, Northwest A&F University, Yangling, Shaanxi, China; 3 Crop Science Group, Institute of Crop Science and Resource Conservation, University of Bonn, Bonn, Germany; University of Western Sydney, AUSTRALIA

## Abstract

Vulnerability assessments have often invoked sustainable livelihoods theory to support the quantification of adaptive capacity based on the availability of capital—social, human, physical, natural, and financial. However, the assumption that increased availability of these capitals confers greater adaptive capacity remains largely untested. We quantified the relationship between commonly used capital indicators and an empirical index of adaptive capacity (ACI) in the context of vulnerability of Australian wheat production to climate variability and change. We calculated ACI by comparing actual yields from farm survey data to climate-driven expected yields estimated by a crop model for 12 regions in Australia’s wheat-sheep zone from 1991–2010. We then compiled data for 24 typical indicators used in vulnerability analyses, spanning the five capitals. We analyzed the ACI and used regression techniques to identify related capital indicators. Between regions, mean ACI was not significantly different but variance over time was. ACI was higher in dry years and lower in wet years suggesting that farm adaptive strategies are geared towards mitigating losses rather than capitalizing on opportunity. Only six of the 24 capital indicators were significantly related to adaptive capacity in a way predicted by theory. Another four indicators were significantly related to adaptive capacity but of the opposite sign, countering our theory-driven expectation. We conclude that the deductive, theory-based use of capitals to define adaptive capacity and vulnerability should be more circumspect. Assessments need to be more evidence-based, first testing the relevance and influence of capital metrics on adaptive capacity for the specific system of interest. This will more effectively direct policy and targeting of investment to mitigate agro-climatic vulnerability.

## Introduction

Crop yields are vulnerable to climatic variability and change [1–6]. While climatic variability is the norm in most agriculturally-important regions [7,8], it is expected to increase, along with the incidence of extreme events such as drought [9,10]. Crop production will need to further adapt through management responses that mitigate yield losses in dry years and capitalize on opportunity in wet years. Regions respond to climatic variability differently, with some regions better able to adapt than others [11]. Quantification of the adaptive capacity of crop production to climatic variability and change at a regional scale through integrating socio-economic indicators is commonly used in the assessment and management of vulnerability [11–16]. The development of reliable, evidence-based indicators that can be used to inform policy and boost the capacity of agriculture to adapt to climatic variability and change is becoming increasingly important [17].

Most previous relevant work on the adaptation of crop production to climatic variability has been framed in terms of crop-drought vulnerability. A plurality of concepts, methods, data, and indicators have been used in this context, reflecting the broader vulnerability field in general [17]. Typically, vulnerability analyses include measures of exposure (degree of disturbance), sensitivity (impact of disturbance), and adaptive capacity (ability to respond to disturbance) [18–20]. Simelton et al. [21] calculated a crop-drought vulnerability index for China’s agricultural regions as the ratio of a wheat failure index and a drought index. They then classified those provinces experiencing less drought impact on crop production as resilient, and those experiencing greater impact as sensitive. A parallel approach was used to assess crop-drought vulnerability and adaptation options represented by socio-economic metrics that emphasized both biophysical and socio-economic adaptation [15]. Antwi-Agyei et al. [12] calculated national crop-drought vulnerability in Ghana as a yield sensitivity index divided by an exposure index, and calculated regional vulnerability as a sensitivity index plus an exposure index, minus an adaptive capacity index. Antwi-Agyei et al. [12] found geographical and socioeconomic patterns in crop-drought vulnerability, and that vulnerable regions have the lowest adaptive capacity.

Vulnerability and adaptive capacity assessments have often been grounded in sustainable livelihoods theory (SLT) [11,22–25]. The SLT states that sustainable livelihoods are achieved through access to a range of livelihood resources—social, human, physical, natural, and financial capital. Indicators are commonly used as proxies for capital types. A wide range of indicators have been used including aspects of demography, population, households, land and environment, technology, health and education, economy, community, labor and employment, socioeconomic development, and livelihood diversification [14,16,26–30]. The livelihood vulnerability index, for example, was calculated as the difference between exposure (e.g. degree of natural disaster) and adaptive capacity (a function of socio-demographics, livelihood strategies, and social networks), multiplied by sensitivity (a function of human heath, access to food, and water) [24].

The underpinning assumption of SLT is that increasing capital in rural communities reduces vulnerability [31]. Many deductive, or *theory-driven*, studies assume that selected capital indicators influence adaptive capacity and vulnerability based on the tenets of SLT [17,32]. Hahn et al. [24] assumed that all capital indicators increased adaptive capacity, and hence, reduced vulnerability. Gbetibouo et al. [23] assumed that increased social capital (share of farmers in farm organizations), physical capital (infrastructure index), human capital (literacy rate), and financial capital (farm income, farm holding size, farm assets, and access to credit) increased adaptive capacity. Gbetibouo et al. [23] also assumed that other aspects of human capital (HIV prevalence) and financial capital (share of people below the poverty line, share of agricultural GDP) reduced adaptive capacity. Nelson et al. [11] quantified adaptive capacity using metrics of human capital (operator education, spouse education, self-assessed health), social capital (land care membership, partners, internet), natural capital (pasture growth index, remnant vegetation), physical capital (plant and machinery, dams, structures, livestock), and financial capital (capital, total cash income, access to finance). Nelson et al. [11] acknowledged that the relationships between capitals are too complex to quantify, and assumed that the five capitals complemented each other in the process of supporting livelihoods.

There have been few inductive, or *data-driven*, studies which have tested this assumption empirically in the context of agro-climatic vulnerability. Nelson et al. [32] and Hinkel [17] have recognized the preponderance of deductive, theory-driven approaches and note that inductive, data-driven examples are scant due to the lack of data on slow-onset hazards such as climate change. Simelton et al. [21] found that a few socio-economic indicators were significantly, but weakly, correlated with an empirical measure of crop vulnerability to drought in China. Similarly, at the global level, Krishnamurthy et al. [33] found significant and strong relationships between hunger and only a few capital-type variables and Fraser et al. [13] found no general statistically significant relationships between seven socio-economic factors and adaptive capacity in cropping systems. Conversely, Simelton et al. [14] found significant correlations between the vulnerability of food crops to climate change and several agro-environmental, governance, and income variables using linear mixed effects models. Despite not explicitly framing the analyses in SLT, these studies demonstrate that empirical relationships between capital-type indicators and vulnerability vary substantially and do not strongly support the underpinning assumptions of theory-driven, deductive applications. Without evidence linking specific elements of capital to adaptive capacity and decreased vulnerability, there is a risk of inefficiency, or even complete failure, of agro-climatic adaptation policy and management strategies based on deductive approaches.

In this study, we explicitly assessed the relationships between common capital indicators and adaptive capacity in Australian wheat production in the context of vulnerability to climatic variability and change. First, we structured the problem into the components of vulnerability: exposure, sensitivity, adaptive capacity. We then developed an empirical metric of vulnerability and adaptive capacity of wheat production for 12 regions in the Australian wheat-sheep zone for the 20 years from 1991–2010. Exposure was represented by annual expected wheat yields calculated for each region using a crop model. Vulnerability was represented as the difference between expected yields and actual yields reported in a farm survey. The data is available in S1 Dataset. We posited that variation in the ratio of vulnerability to exposure—the relative performance of actual versus expected wheat yields—was a function of the sensitivity and adaptive capacity of regions to climatic variability. We then selected 24 commonly used indicators of social, human, physical, natural, and financial capital and assembled spatio-temporal data from a range of sources. A sequence of regression techniques and supporting statistical tests were used to robustly identify those capital indicators driving variance in the empirical measure of vulnerability and thereby, significantly related to adaptive capacity. We discuss the implications for reducing the agro-climatic vulnerability of Australian wheat production, and for deductive, theory-driven application of SLT in vulnerability studies.

## Methods

### Study area

**Fig 1:**
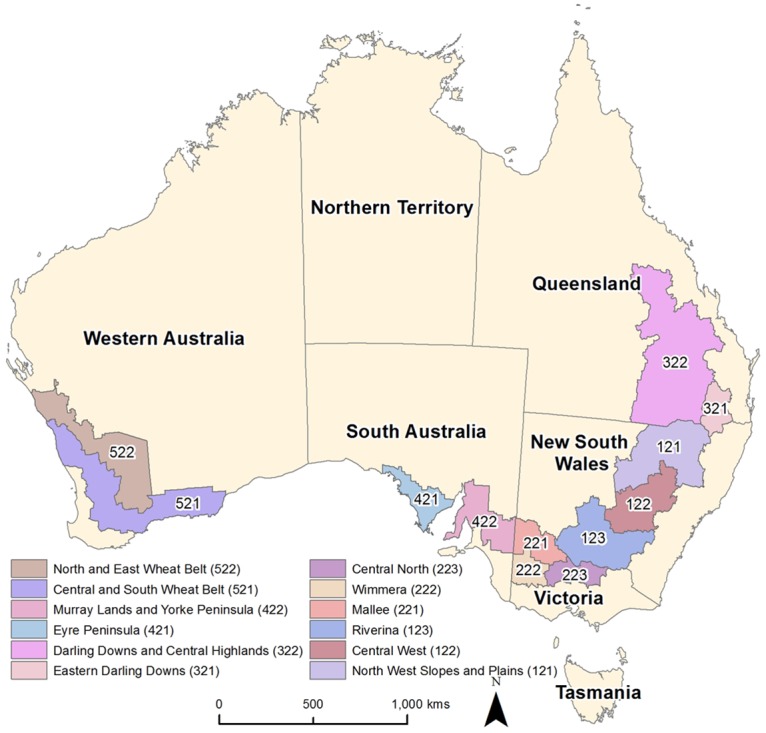
Study area. Location of the 12 ABARES regions in the wheat-sheep zone of Australia.

Wheat is the most widely grown crop globally, and in Australia. Exported to over 40 countries, Australian wheat plays an important role in global food security. In Australia, total wheat production in 2006 was just over 25 million tonnes from 12.443 million ha, with about 90% produced in the wheat-sheep zone—our study area (Fig. 1). We used the 12-region Australia Bureau of Agricultural and Resource Economics and Sciences (ABARES) classification as the geographical basis for analysis in this study (Fig. 1) because of the availability of data on actual yields and capital indicators. The average area of each region was 9.144 million ha.

The study area has experienced increased climatic variability and change, and this has been observed to affect wheat yields. Much of this zone has experienced decreasing rainfall with much of this decline occurring in autumn (March—May). Daily maximum and minimum temperatures are also rising [34]. From 2006–2007, droughts related to El Niño events caused dramatic decreases in rainfall in Australia’s wheat-sheep zone and caused wheat production to fall by roughly 61% in the study area [35].

### Conceptualizing vulnerability

Vulnerability (*V*) is commonly conceptualized as a function of exposure (*E*), sensitivity (*S*), and adaptive capacity (*AC*) [18]. In the context of agro-climatic vulnerability, exposure refers to the nature and degree to which an agricultural system is subject to significant climatic variation and change [36]. Sensitivity reflects the response of the system to climatic variation and change, either positively or negatively, and may be influenced by socio-economic and environmental factors [18]. Adaptive capacity refers to the ability to adapt to the impacts of climatic variation and change [37]. In simple terms, vulnerability is positively related to exposure and sensitivity, and negatively related to adaptive capacity. Hence, we may represent it as:

$$V=f\left(\frac{E\times S}{AC}\right)$$ 1

Rearranging equation 1 we can represent the ratio of vulnerability and exposure as a function of sensitivity and adaptive capacity:

$$\frac{V}{E}=f\left(\frac{S}{AC}\right)$$ 2

We let expected wheat yield, calculated using a crop model, represent exposure to climatic variability. The difference between actual and expected wheat yield provides an empirical (also could be termed *observed* or *revealed*) measure of vulnerability. The term *V*/*E* in Equation 2 thereby quantifies the relative performance of actual wheat yields against expected. The function of sensitivity and adaptive capacity *f*(*S*/*AC*) incorporates those unobserved characteristics of agricultural regions that explain the variance in the ratio of vulnerability and exposure—or, in other words, why regional annual wheat production performs better or worse than expected. It captures those factors that determine the sensitivity of wheat production to climatic variability and change, and its capacity to adapt. These characteristics determine whether wheat production is sensitive to poor seasons (i.e. dry years) or able to adapt management strategies to mitigate yield losses. They also determine whether wheat production can adapt management strategies to capitalize on opportunity in good seasons (i.e. wet years). Hereafter, we simply refer to this broadly as *adaptive capacity* and the term *f*(*S*/*AC*) as the adaptive capacity index. Under the SLT framework [38], these characteristics consist of social, human, physical, natural, and financial capital. Based on SLT, we hypothesized that better access to these five capitals confers greater adaptive capacity and can thereby reduce vulnerability to climate variability and change in Australian wheat farming.

### Empirical indicators of vulnerability and adaptive capacity

Exposure was represented by an expected wheat yield index calculated using modeled yields from the Agricultural Production Systems Simulator [APSIM, 39]. APSIM has been extensively verified [40,41] and used to simulate agricultural systems [42–45]. We used APSIM version 7.3 to simulate wheat yield in an annual, continuous wheat system from 1991–2010 with a model spin-up period from 1900–1990. We used a fixed fertilization rate of 50 kgN ha^-1^, with 25 kgN ha^-1^ applied at sowing and 25 kgN ha^-1^ top-dressed at stem elongation. We assumed minimum tillage techniques where 100% of crop residue was retained on the soil surface. Typical sowing windows and cultivars were selected for each state and these allow selection of early or late cultivars based on rainfall timing (Table 1). No weed control was assumed between crops and wheat was harvested at maturity [46].

**Table 1:** Sowing windows and cultivars used in APSIM modeling.

Location	Consecutive rainfall	Cultivars and sowing windows
QLD	25mm in 10 days	Janz (10 May-30 Jun), Hartog (1 Jul-30 Jul)
NSW	25mm in 10 days	Batavia (20 Apr-30 Apr), Sunco (1 May-31 May), Buckly (1 Jun-30 Jun), Hartog (1 Jul to 30 Jul)
VIC	16mm in 6 days	Sunco (1 May-31 Apr), Buckly (1 Jun-30 Jun)), Hartog (1 Jul to 30 Jul)
SA	25mm in 10 days	Batavia(20 Apr-30 Apr), Sunco (1 May-31 May), Buckly (1 Jun-30 Jun), Hartog (1 Jul to 30 Jul)
WA	25mm in 10 days	Spear (20 Apr-31 May), Kulin (1 Jun-15 Jul)

Wheat yield was simulated for 6189 spatial climate—soil (CS) units across the study area. For each CS unit we summarized daily gridded (5km grid cell resolution) climate data [47] including temperature (max, min), rainfall, solar radiation, and potential evapotranspiration [48]. Soil profile data was derived from the Australian Soil Resources Information System (ASRIS) [49]. Modeled wheat yields were masked by the area mapped as wheat in the National Land Use Map of Australia 2006 [50], and averaged spatially over each region for each year.

The expected wheat yield index $WYI_{rt}^{Exp}$ was then calculated as a normalized yield anomaly index—the ratio of the expected wheat yield (t ha^-1^ yr^-1^) for each region *r* and year *t* ($WY_{rt}^{Exp}$) to the average expected wheat yield (t ha^-1^ yr^-1^) for each region over all years (${\bar{WY}}_{r}^{Exp}$) such that $E_{rt}=WYI_{rt}^{Exp}=WY_{rt}^{Exp}/{\bar{WY}}_{r}^{Exp}$.

The empirical indicator of vulnerability was calculated using the expected wheat yield index (exposure) and an actual wheat yield index. The actual wheat yield index $WYI_{rt}^{Act}$ was derived from wheat yields reported in ABARES Australian Agricultural and Grazing Industries Survey [AAGIS; 51]. AAGIS is conducted each financial year and contributes to the AgSurf database which includes data for the last twenty years on farm performance, production benchmarks, farm management, and socio-economic indicators relating to the grains, beef, sheep, and dairy industries in Australia. The AgSurf data has been widely used to assess Australian farming systems [11,52]. Farm surveys averaged 66 respondents (SD = 25) from each region each year from an average farmer population of 3219 (SD = 1427).

$WYI_{rt}^{Act}$ was also calculated as a normalized yield anomaly index—the ratio of the actual wheat yield (t ha^-1^ yr^-1^) for each region *r* and year *t* ($WY_{rt}^{Act}$) and the average actual wheat yield (t ha^-1^ yr^-1^) for each region over all years (${\bar{WY}}_{r}^{Act}$) such that $WYI_{rt}^{Act}=WY_{rt}^{Act}/{\bar{WY}}_{r}^{Act}$. Informed by previous analyses [53], we considered that no systematic productivity increase occurred over the period of study. Both the actual and expected wheat yield indices have an average of 1.

Vulnerability (*V*) was calculated as the difference between the actual and expected yield indices such that $V_{rt}=WYI_{rt}^{Act}-WYI_{rt}^{Exp}$. The adaptive capacity index *ACI*_*rt*_ was then calculated as the ratio of vulnerability to exposure as per Equation 2:

$$ACI_{rt}=\frac{V_{rt}}{E_{rt}}=\frac{WYI_{rt}^{Act}-WYI_{rt}^{Exp}}{WYI_{rt}^{Exp}}$$ 3

In Australian cropping regions, adaptive responses to climatic variability are being encouraged which match inputs to yield potential and respond to information such as soil moisture at sowing, break of season timing, crop simulations (e.g. Yield Prophet http://www.yieldprophet.com.au), and seasonal forecasts. Reducing inputs in poor seasons and increasing inputs in good seasons is a key adaptation at the farm level. Effective adaptation would manifest as $WYI_{rt}^{Act}\approx WYI_{rt}^{Exp}$ and $ACI_{rt}\approx 0$. A negative value of *ACI*_*rt*_ indicates lower than expected yields reflecting lower adaptive capacity, while a positive value indicates better than expected yields and greater adaptive capacity.

In exploring the *ACI* metric, we used ANOVA to test whether the mean *ACI* score differs between regions. We also used *t*-tests to test for significant difference in mean *ACI* between dry years ($WYI_{rt}^{Exp}<0$) and wet years ($WYI_{rt}^{Exp}\geq 0$), both over all regions, and for each region individually.

### Capital indicators

Based on Ellis [38], Nelson et al. [11] and the availability and quality of data, we identified 24 indicators to represent adaptive capacity (Table 2). Based on our judgment of best fit, we classified each indicator as one of five specific types of capital—social, human, physical, natural, and financial. While this classification is imperfect, often because the distinction between capitals is fuzzy and indicators could adequately represent more than one capital, misclassification is inconsequential. We hypothesized that greater access to capital as represented by these proxy indicators increases adaptive capacity and reduces the climatic vulnerability of Australian wheat farming.

**Table 2:** Capital indicator labels, descriptions, units of measurement, summary, and source.

Indicator	Variable name	Definitions	Unit	Estimate 5th, 50th, 95th perc	Standard error^a^ (%) 5^th^, 50^th^, 95^th^ perc	Source
Social capital						
Ownership	Family share of farm income (SC_FSFI)	Ownership share of farm income of owner manager, spouse and dependent children during the survey year. Farm income was defined as total cash income or the difference between total cash receipts and total cash costs.	$	-32373, 15710, 79864	18, 43, 958	AgSurf
Communication	Telephone charges (SC_Phone)	Total telephone expenses incurred during the survey year averaged per farm	$	1459, 2433, 4211	7, 11, 19	AgSurf
Remoteness^b^	Accessibility and Remoteness (SC_Remote)	Accessibility and Remoteness Index of Australia. Spatial index of road distance to between populated localities and population/service centers of various sizes (low values = more accessible, high values = more remote)	Score	-26, 5, 10	NA	GISCA
Human capital						
Information access	Advisory services (HC_Advis)	Total fees paid for advisory services such as farm consultants during the survey year	$	70, 578, 2916	22, 42, 83	AgSurf
Diversification	Crop diversity (HC_CropDiv)	Diversity of average area per farm sown to different crops during the survey year. Calculated using the Gini-Simpson diversity index $1-\lambda =\;1-\overset{R}{\underset{i=1}{\sum }}p_{i}{}^{2}$ where $p_{i}{}^{2}$ is the proportion of the average area per farm of crop *i* and *R* is the set of crops: barley, grain legumes, oats, oilseeds, rice, sorghum, and wheat. 1- λ ranges from 0 (no diversity, only one crop grown) to 1–1/*R* (maximum diversity, equal proportions of all crops grown)	Index (0–0.857)	0.44, 0.63, 0.76	10, 25, 73	AgSurf
	Livestock diversity (HC_LSDiv)	Average livestock herd at 30 June per farm calculated as the beef herd size plus sheep flock size converted to dry sheep equivalent units (DSE) where 1 head beef = 10 DSE, 1 head sheep = 1.5 DSE	DSE	1627, 3278, 8103	8, 17, 64	AgSurf
Workforce	Total labor used (HC_Labor)	Average total number of full time weeks worked by all farm workers including hired labor per farm during the survey year. If an individual works less than 40 hours in an average week, the estimate is converted into a full time week equivalent	# full time weeks	81, 103, 127	4, 6, 11	AgSurf
Education^b^	Education and occupation (HC_Educ)	SEIFA (Socio-Economic Indexes for Areas) index of education and occupation which combines census (2006) variables relating to the educational and occupational characteristics of communities, such as the proportion of people with a higher qualification or those employed in a skilled occupation (low values = more disadvantaged, high values = more advantaged)	Score	937, 984, 1022	NA	ABS^c^
Physical capital						
Structures	Value of land and fixed improvements (PC_ValLI)	Estimate of the market value of all land operated and fixed improvements as of the end of the financial year, which was estimated by the survey respondent	$	862685, 1705496, 4335848	6, 9, 16	AgSurf
Infrastructure	Electricity use (PC_Elec)	Average expenditure per farm on electricity during the survey year	$	1239, 2543, 4153	9, 13, 25	AgSurf
Intensity of inputs	Fertilizer use (PC_Fert)	Average expenditure per farm on crop and pasture chemicals and soil conditioners during the survey year	$	4953, 26561, 122520	8, 14, 29	AgSurf
	Chemical use (PC_Chem)	Average expenditure per farm on fertilizers during the survey year	$	5301, 19805, 77065	9, 15, 27	AgSurf
	Fuel use (PC_Fuel)	Average expenditure per farm on fuel oil and grease during the survey year	$	11929, 23932, 45707	7, 11, 19	AgSurf
Land	Area cropped (PC_Land)	Total farm area cropped (total area of crops sown or planted less areas double counted or inter-planted) including areas cut for hay	ha	150, 405, 1559	5, 10, 17	AgSurf
Natural capital						
Climate	Maximum temperature (NC_MaxT)	Maximum temperature in the period from sowing to harvest in the APSIM simulations	Deg. C	16, 19, 25	NA	BOM^d^
	Gowing season rainfall (NC_SHRain)	Total rainfall in the period from sowing to harvest in the APSIM simulations	mm	90, 233, 410	NA	BOM^d^
	Total rainfall (NC_TRain)	Total rainfall in the calendar year of the crop simulation	mm	234, 386, 639	NA	
	Solar radiation (NC_SRad)	Accumulated solar radiation in the period from sowing to harvest in the APSIM simulations	MJ m^-2^	2294, 2614, 2924	NA	BOM^d^
Soils^b^	Soil water holding capacity (NC_SWHC)	Drained upper limit minus crop lower limit averaged over the zone. Drained upper limit was defined as the amount of water that a particular soil holds after drainage has practically ceased. Crop lower limit was defined as the amount of water remaining after a particular crop has extracted all the water available to it from the soil	mm mm^-1^	0.10, 0.13, 0.17	NA	ASRIS^e^
Native vegetation^b^	Native vegetation (NC_NVeg)	Percentage of each region covered by native vegetation. Native vegetation was classified as native forests, woodlands, shrublands, heathlands, grasslands, and minimally modified pastures in the dynamic land cover database from 2008	%	18, 41, 92	NA	GA^f^
NPP^b^	Net primary productivity (NC_NPP)	Mean annual net primary production data from MODIS (MOD17A3) data from 2000–2009.	(t ha^-1^ year^-1^)	2.94, 3.97, 7.33	NA	NTSG^g^
Financial						
Capital	Total closing capital (FC_TCCap)	The closing value of all assets used on the farm including leased equipment but excluding machinery and equipment either hired or used by contractors based on market value of land and fixed improvements and livestock/crop inventories and replacement value less depreciation for plant and machinery	$	1079689, 2156772, 5320751	5, 8, 15	AgSurf
Access to finance	Access to credit (FC_AccCred)	Access to credit equals the sum of borrowing capacity and liquid assets. Borrowing capacity was derived according to each farm’s equity ratio. Where the equity ratio is less than 70 per cent, borrowing capacity is zero, otherwise borrowing capacity = (equity ratio − 0.70) × total closing capital (see above)	$	205282, 460927, 1006449	2, 9, 36	AgSurf
Income	Recent family income (FC_RFInc)	Income level in year *n* was calculated as the 5-year moving average (years *n*-1 to *n*-5) of total family income calculated as the family share of farm income plus all off-farm income of owner manager and spouse. It is the amount of income available to households to meet living and other expenses	$	23684, 46668, 70012	14, 28, 256	AgSurf

^a^ Standard error as reported for the AgSurf survey data only

^b^ Indicates time-invariant data (i.e. varies by region only)

^c^ Australian Bureau of Statistics

^d^ Bureau of Meteorology

^e^ Australian Soil Resources Information System

^f^ Geoscience Australia

^g^ Numerical Terradynamic Simulation Group, University of Montana

Social capital metrics included farm ownership and communication which, we hypothesized, increased adaptive capacity. Higher family share of farm income reflects a sense of ownership of farm production. Farmers with a greater share of farm income were posited to employ more managerial and technical skills in adapting farm production to climatic variability. Telephone expenditure provided a proxy for communication and social networks. Greater sharing of land management knowledge and practices between land managers through social networks can provide *win-win-win* benefits of reducing system vulnerability, increasing income, and building social capital [54]. Remoteness—the third measure of social capital—we hypothesized, reduced adaptive capacity through less access to peer networks and information, and a range of other physical, human, and financial capital.

Human capital included advisory services, diversification, workforce, and education. Access to advisory services can provide information such as forecasts and contextualized management advice. Crop and livestock diversification reflects the diversity of the farm business and indicates greater risk management [42]. Access to labor can increase the ability to adapt to climatic variability through providing the workforce and know-how to implement management responses. Higher education levels can reflect increased technical skill of the farm manager and also the service centers within a region.

Physical capital was represented by the presence of structures, infrastructure, intensity of inputs, and land resources which we hypothesized to increase adaptive capacity. The presence of structures of greater value determined by the value of land and improvements suggests that farmers have more access to technology which helps improve crop yield, offsetting the possible negative impacts of climatic variability and change [55,56]. Access to infrastructure such as electricity use provides resources for and equips management responses [57,58]. Greater intensity of inputs (i.e. fertilizer, chemicals, fuel) is also likely to increase adaptive capacity to climatic variability. For example, increased use of fertilizers can restore depleted soil nutrients and increase crop yields [59,60]. Greater access to land for cropping reflects increased area of higher quality land available for production, and the ability to spread risk of localized disturbances over a larger area.

Natural capital included a range of climatic variables (maximum temperature, growing season rainfall, total rainfall, and solar radiation), soil water holding capacity, the presence of native vegetation, and net primary productivity. Climatic variables directly influence crop yield. Good soil water holding capacity can increase water availability to crops, enhance growth, and facilitate plant persistence during drought [61]. The presence of native vegetation influences the provision of ecosystem services such as the mitigation of erosion and soil salinization that directly contribute to crop production [62,63]. Net primary productivity is also directly related to crop growth.

Financial capital was represented by total closing capital, access to credit, and income level. We hypothesized that high total closing capital conferred greater adaptive capacity because wealth provides better access to markets, technology, and other resources that can be used to adapt to climatic variability and change [64,65]. Better access to credit enables farmers to borrow money to reduce financial risk, buffer variation in income, and to access technology. Higher income levels also increase adaptive capacity through the increased ability to invest in technology such as machinery, new crop varieties, and inputs.

### Testing the explanatory power of capital indicators

We assessed the influence of the capital indicators as explanatory variables on the empirical index of adaptive capacity using a sequence of statistical tests undertaken in STATA. First, we undertook a standard linear regression with random regional effects such that the term *f*(*S*/*AC*) in Equation 2, represented by the adaptive capacity index *ACI*, was estimated using the standard linear model:

$$f\left(\frac{S}{AC}\right)=ACI=C+\beta X+\varepsilon$$ 4

where *C* is a constant term, **X** is a vector of all 24 explanatory capital variables, and **β** is a vector of coefficients for each capital variable, and *ε* is an error term.

The initial linear regression returned an *R* ^*2*^ of 0.3916 with five statistically significant explanatory capital variables (*α* = 0.05) (S1 Table). We tested for heteroscedasticity (variance of residuals changing with explanatory variables) using a Breusch-Pagan / Cook-Weisberg test [66]. A high chi-square value (*χ* ^2^ = 264.33, *P* > χ^2^ = 0.000) indicated prevalence of heteroscedasticity which we dealt with using heteroscedasticity-robust standard error regression.

We then tested for effects of time-invariant omitted variables associated with each region using fixed effects regression to carry out *t*-tests on regional dummy variables with the model:

$$f\left(\frac{S}{AC}\right)=ACI=C+\beta X+\alpha +\varepsilon$$ 5

Here, *C* is again a constant term and *ε* is the error, **X** is a vector of all 24 explanatory capital variables, and **β** is a vector of coefficients for each variable in **X**. *α* is a constant term unique to each region and captures region-specific, unobserved, time-invariant factors affecting *ACI* (termed the *unobserved* or *fixed effect*). Significant effects were detected (*α* = 0.05) for seven of the 12 regions. In addition, the regression with fixed effects performed better than the standard linear regression, yielding a higher *R* ^*2*^ of 0.4441 and identifying seven significant explanatory capital variables (*α* = 0.05) (S2 Table). Hence, we used fixed effects regression to treat the omitted variable bias associated with regional effects.

Significant results from the Ramsey Regression Equation Specification Error Test [RESET, 67] suggested that non-linear functional forms for capital variables may improve explanatory power. We explored alternative functional forms for both the dependent and explanatory variables using the *gladder* procedure which searches a subset of the ladder of powers [68] for a transformation that best converts each variable to a normal distribution. Results identified appropriate transformations for 14 variables (S1 Supporting Information). Variable transformation improved the explanatory power with *R* ^*2*^ = 0.5625 and nine significant capital variables (*α* = 0.05) (S3 Table).

Finally, multicollinearity was assessed and confounding variables were identified using a variance inflation factor (VIF) test. Of the 12 variables with a VIF > 10 (S4 Table), we removed six (HC_LSDiv, PC_VLFI, PC_Chem, PC_Fuel, NC_MaxT, FC_TCCap) from the model whose explanatory power was not significant (*α* = 0.01). We then ran the final regression model. A subsequent VIF test for multicollinearity identified five remaining explanatory variables with VIF > 10 which we left in the model as they were both highly significant and conceptually important. This remnant multicollinearity will inflate the reported standard errors but will not affect the conclusions of the analysis.

## Results

### Temporal and spatial patterns in empirical indicators

**Fig 2:**
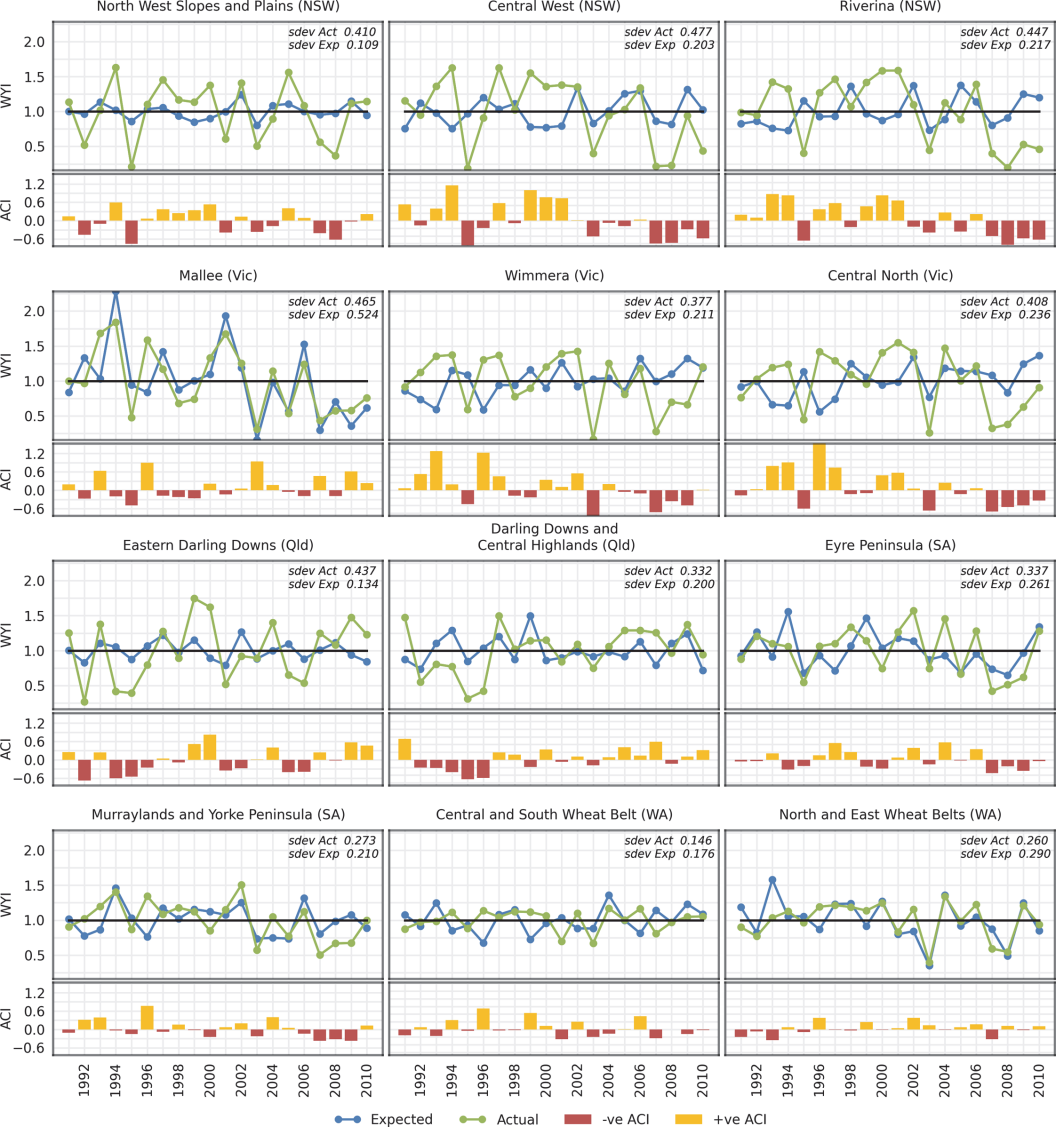
Wheat yield and adaptive capacity indices. Actual and expected wheat yield indices and adaptive capacity index for each region from 1991–2010.

The actual and expected wheat yield indices (WYIs) ranged from 0.170 to 1.841, and from 0.161 to 2.286, respectively (Fig. 2). Overall, the actual WYI was more variable (SD = 0.367) than the expected (SD = 0.246). Variance in actual and expected WYI differed between regions (Fig. 2). For example, North West Slopes and Plains (NSW) and Eastern Darling Downs (Qld) had low variance in expected WYI but a high variance in the actual WYI. Mallee (Vic) had high variance in both expected and actual WYI, with actual WYI tracking closely to expected. Actual WYI also tracked closely to expected in the two WA and SA regions, with Central and South Wheat Belt (WA) exhibiting low variance in both actual and expected WYI.

**Fig 3:**
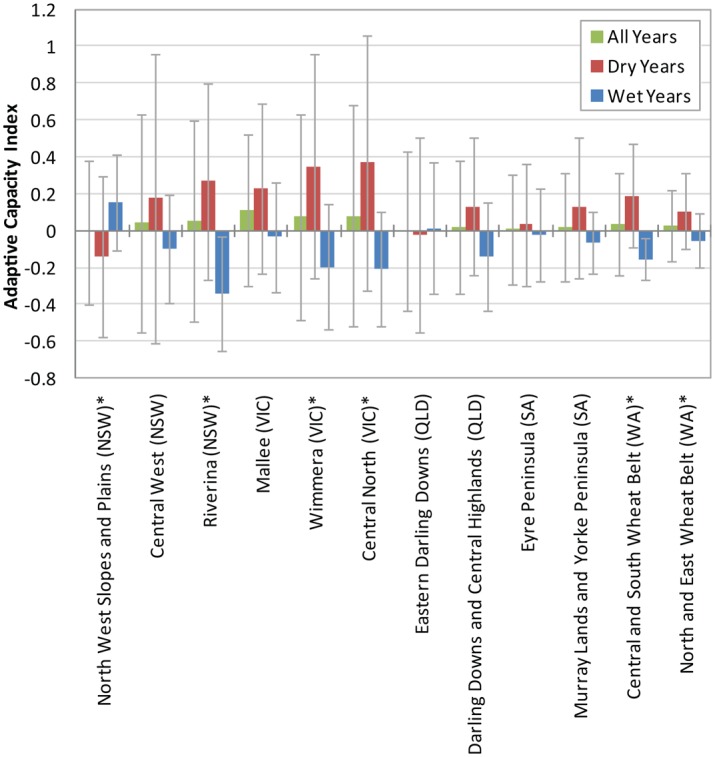
Adaptive capacity index for wet and dry years. Mean and standard deviation of adaptive capacity index by region for all years, wet years (where expected WYI^Exp^ ≥ 1), and dry years (where expected *WYI* ^*Exp*^ < 1). * indicates significant difference in ACI between dry and wet years (*α* = 0.1).

Mean ACI over all regions and years was positive but low (mean = 0.038) and highly variable (SD = 0.424). In most regions, the ACI tends to exhibit runs of a few years with positive or negative ACI, with a noticeable run of negative ACI years coinciding with drought in the late 2000s (Fig. 2). On average, most regions had a near zero but positive ACI with Mallee (Vic) the highest (mean ACI = 0.112) (Fig. 3). There was no significant difference in mean ACI between regions (*P* = 0.9995) but the variance did differ significantly between regions (*P* < 0.000). The four WA and SA regions had the lowest variance, particularly North and East Wheat Belt (WA) (SD = 0.192). ACI was significantly higher (*P* < 0.000) in dry years (mean ACI = 0.152, SD = 0.490) than in wet years (mean ACI = -0.092, SD = 0.283) overall, and significant for six of the 12 regions (*α* = 0.1) (Fig. 3).

### Determinants of adaptive capacity

The explanatory power of the final regression model (*R* ^*2*^ = 0.5470) with the reduced variable set was slightly lower than the model with the full set (*R* ^*2*^ = 0.5625) but with the advantage of reduced multicollinearity (S5 Table) and lower standard errors (Table 3). A total of 10 capital variables were significantly related to the empirical measure of adaptive capacity including one social capital variable (SC_Phone), one human capital variable (HC_Educ), two physical capital variables (PC_Fert, PC_Land), six natural capital variables (NC_SHRain, NC_Train, NC_SRad, NC_SWHC, NC_NVeg, NC_NPP), and no financial capital variables. However, of these 10 variables, four displayed coefficients of the opposite sign to that predicted by theory (SC_Phone, HC_Educ, PC_Land, NC_SWHC).

**Table 3:** Results of the final fixed effects regression analysis.

Capital	Description	Code	Transform	Expectation	Coef.	Robust Std. Err	t	P>|t|	[95% Conf. Interval]	
Social	Family share of farm income	SC_FSFI	-		6.16E-07	6.45E-07	0.96	0.341	-6.56E-7	1.89E-06
	Telephone charges	SC_Phone	Inv. sqrt	–	50.36992	15.7503	3.2	0.002	19.32515	81.41469
	Accessibility and Remoteness	SC_Remote	Inverse		-0.49933	0.3465744	-1.44	0.151	-1.18245	0.183791
Human	Advisory services	HC_Advis	Sqrt		0.003471	0.002235	1.55	0.122	-0.00093	0.007876
	Crop diversity	HC_CropDiv	-		0.304657	0.4048501	0.75	0.453	-0.49333	1.102641
	Total labor used	HC_Labor	-		-0.00058	0.0027764	-0.21	0.834	-0.00605	0.00489
	Education and occupation	HC_Educ	-	–	-0.00407	0.0017318	-2.35	0.020	-0.00748	-0.00065
Physical	Electricity use	PC_Elec	-		3.89E-05	0.0000447	0.87	0.385	-4.9E-05	0.000127
	Fertilizer use	PC_Fert	Log	+	0.37311	0.0850215	4.39	0.000	0.205528	0.540692
	Area cropped	PC_Land	Log	–	-0.51106	0.136713	-3.74	0.000	-0.78053	-0.2416
Natural	Gowing season rainfall	NC_SHRain	-	+	0.002167	0.0004953	4.37	0.000	0.001191	0.003143
	Total rainfall	NC_TRain	-	+	0.001149	0.0003908	2.94	0.004	0.000379	0.001919
	Solar radiation	NC_SRad	-	+	0.000459	0.0002305	1.99	0.048	4.71E-06	0.000913
	Soil water holding capacity	NC_SWHC	-	–	-87.0139	20.55461	-4.23	0.000	-127.528	-46.4996
	Native vegetation	NC_NVeg	Log	+	1.399986	0.364436	3.84	0.000	0.681661	2.118311
	Net primary productivity	NC_NPP	Inverse	+	-34.5693	8.929121	-3.87	0.000	-52.1691	-16.9695
Financial	Access to credit	FC_AccCred	Log		0.098395	0.0652794	1.51	0.133	-0.03027	0.227064
	Recent family income	FC_RFInc	-		2.11E-06	1.57E-06	1.35	0.180	-9.80E-7	5.20E-06
	Region									
	Central West (NSW)				-1.28521	0.3320974	-3.87	0.000	-1.93979	-0.63062
	Central and South Wheat Belt (WA)				-4.31125	1.040959	-4.14	0.000	-6.36304	-2.25946
	Darling Downs & Cent. Highlands (QLD)				-6.02779	1.748972	-3.45	0.001	-9.47512	-2.58046
	Eastern Darling Downs (QLD)				-4.68966	1.234197	-3.8	0.000	-7.12234	-2.25699
	Eyre Peninsula (SA)				-1.54657	0.5015465	-3.08	0.002	-2.53515	-0.55799
	Mallee (VIC)				0.536651	0.1338944	4.01	0.000	0.272738	0.800565
	Murral Lands and Yorke Peninsula (SA)				0					
	North West Slopes and Plains (NSW)				0					
	North and East Wheat Belt (WA)				0					
	Riverina (NSW)				0					
	Wimmera (VIC)				0					
	Constant				14.30915	4.342876	3.29	0.001	5.749086	22.86922

Capital variables with significantly related to the empirical adaptive capacity index (*α* = 0.05) are in bold. Note that the base region to which all other regions are compared is Central North (VIC), with the intercept for Central North (VIC) equal to the constant term. Positive expectation indicates that the sign of the coefficient is consistent with theory, negative indicates that a result counter to that expected by theory given the transformation applied to the independent variables (noting the specific value reversal effect of inverse transformation).

## Discussion

We calculated empirical indicators of the vulnerability of Australian wheat production to climatic variability over the 20 years from 1991–2010. Our approach involved calculating an expected yield index—an annual anomaly in modeled yield, and an actual yield index—an annual anomaly in survey-reported yield. Exposure of wheat yield to climatic variability was represented by the expected yield index. Vulnerability was represented as the difference between actual and expected yield indices. Adaptive capacity was represented as a function of the ratio of vulnerability to exposure—or the relative performance of actual yields as a proportion of expected yields. The actual and expected yield indices were not perfectly correlated. All regions exhibited years where actual wheat yield was greater than expected and years when low yields were realized relative to expected. We posited that this variation is due at least in part to variation in adaptive capacity between regions and over time. In part, it is also driven by uncertainty in both modeled and survey-reported yields, and other factors that are not accounted for in our data and model such as: subregional variation in biophysical parameters; actual farm management strategies (e.g. sowing date, nature and timing of fertilization and chemical use), and; pest and disease pressures. We found that the mean ACI did not vary between region but the variance in ACI did. ACI also varied significantly over time, with regions tending to have runs of a few years with lower yields than expected, or higher yields than expected. The finding that actual yields tended to be higher than expected in dry years, and lower than expected in wet years is consistent with typical management strategies of Australian farmers. Farmers, experienced and adept at managing in dry conditions, tend to achieve better yields in dry years, while in wet years they tend to under-apply inputs such as fertilizer to mitigate financial risk, resulting in underperformance [69].

We also constructed a set of 24 indicators of regional social, human, physical, natural, and financial capital of the type commonly employed in deductive, theory-driven vulnerability assessments founded in SLT. Consistent with Simelton et al. [21], [13] and Krishnamurthy et al. [33], but in contrast to Simelton et al. [14], we found that few capital indicators were related to the empirical metric of adaptive capacity in a way predicted by theory. Through a robust sequence of regression and supporting statistical tests, we were able to identify significant relationships between the empirical metric of adaptive capacity (*ACI*) and 10 capital indicators. For the remaining 14 capital indicators, no statistically significant relationship to adaptive capacity could be established. Notably however, we could only verify the theory-predicted and *a priori* expected sign of the relationship with adaptive capacity for six of the 10 significant capital indicators. Specifically, we found adaptive capacity significantly and positively related to one physical capital variable fertilization (PC_Fert), and five natural capital variables: growing season rainfall (NC_SHRain), total annual rainfall (NC_TRain), solar radiation (NC_SRad), the presence of native vegetation (NC_NVeg), and net primary productivity (NC_NPP). This supports our earlier hypotheses, that these six variables that may confer adaptive capacity by directly influencing crop growth and, in the case of NC_NVeg, by supporting other ecosystem services that enhance crop yields. The other four significant capital indicators were negatively related to adaptive capacity—countering our theory-based expectations. These included telephone expenditure (SC_Phone), education (HC_Educ), cropped area (PC_Land), and soil water holding capacity (NC_SWHC). In other words, regions spending less time on the telephone, with lower education levels, cropping less land, and with soils capable of holding less water, had greater adaptive capacity. Several potential explanations exist including: Type I statistical errors; capital indicators affect adaptive capacity in non-intuitive ways or may be correlated with some other form of unaccounted-for but influential capital; or *ecological fallacy* resulting from inference about individuals from regionally aggregated data.

There are several limitations to the data used in our study and these will have influenced the results. While our empirically-based vulnerability indicator combined two of the best and most widely used agricultural information sources in Australia—they both have limitations. The ABARE AgSurf farm survey data used to quantify actual wheat yields include low sample sizes relative to the large spatial extent and diverse farming systems of each region, resulting in substantial uncertainty (Table 2). The APSIM modeling used to represent potential yields was of much higher spatial resolution [49] but suffered from a lack of detailed knowledge of the actual agricultural management practices used (i.e. fertilization rates, sowing date etc.). To remove any resulting systematic bias in the vulnerability index, we held management practices (i.e. fertilization rates) constant at typical levels in APSIM, leaving only the climate signal, and to a lesser extent, crop variety [70] as the main effect on potential yield. Deviation from the mean was used to normalize both wheat yield indices. Capital indicator data also varied in quality and resolution. A similar critique of the AgSurf yield data also holds for the AgSurf capital data. Five other data layers were time-invariant (Table 2) such that they captured between-region effects only at a single point in time. Climate data were of high quality and resolution but also suffered from generalization to regional level. The data quality and resolution issues described above are typical of national scale analyses. The negative impact on the results has been minimized where possible and while some uncertainty remains, it is unlikely to change our conclusions.

Our results lead us to draw somewhat different conclusions about regional vulnerability and adaptive capacity in Australian farming than previous deductive studies that built indices of adaptive capacity based on the general logic of SLT but did not test whether capital measures influenced observed vulnerability. For example, the index of vulnerability of rural communities in Australia constructed on SLT logic by Nelson et al. [11] designated parts of SA and WA to be in the 33% most vulnerable to climate change. However, our modeling suggested that these regions were amongst the most adaptive with actual yields tracking expected yields fairly closely, and this conclusion is supported by empirical evidence [71]. The discrepancy may be partly due to differences in our formulation of vulnerability compared to Nelson et al. [11]. More significantly however, some of the measures included in the adaptive capacity index of Nelson et al. [11] may not relate as strongly to observed vulnerability and adaptive capacity as might be supposed by deductive logic.

This finding suggests that caution and further empirical effort should be exercised in applying the SLT in assessing adaptive capacity and vulnerability. The approach is growing in popularity and we concur that it is an effective way to organize thinking about how endowments of multiple forms of capital may influence vulnerability and adaptive capacity of households, farms, and regions to disturbances such as climatic variability and change [20]. However, to date, the predominant practice in SLT-based analyses has been to build indices of vulnerability and adaptive capacity based on general theories about how various types of social, human, physical, natural, and financial capital are likely to relate to adaptive capacity and vulnerability. While this approach seems logical, we failed to find empirical support for it. Our contention is that greater effort is needed in the process of selecting capital indicators in SLT-based vulnerability analyses to empirically test whether the indicators actually relate to vulnerability and adaptive capacity. Absent such efforts, conclusions regarding factors or regions to address to enhance adaptive capacity and reduce vulnerability may be misguided and resources may be allocated where they have little impact.

## Conclusion

Common practice in vulnerability assessments involves constructing indices of social, human, physical, natural, and financial capital measures assumed to affect adaptive capacity based on the deductive logic of sustainable livelihoods theory (SLT), and then using the indices to identify vulnerable regions. To date, however, there has been little testing of the assumption that capitals influence adaptive capacity in the assessment of agro-climatic vulnerability using the SLT. We developed an empirical measure of vulnerability and adaptive capacity based on indices of actual and expected wheat yield for 12 regions in the wheat-sheep zone of Australia from 1991–2010. We then assembled data for 24 capital indicators and statistically tested their effectiveness in explaining adaptive capacity. Only six of 24 indicators were significantly related to adaptive capacity in ways predicted by theory, four were significantly related but with the opposite sign to what would be predicted by theory, and the other 14 were unrelated. We conclude that the use of SLT should be more circumspect and that further empirical work is needed on the selection of capital indicators which have an empirically-established relationship with adaptive capacity. Failure to do this may result in misguided policy and targeting of investment for increasing adaptive capacity and reducing vulnerability to climate variability. While we concur that SLT is a useful construct, we believe that there is still significant work required to make it operationally useful. In agro-climatic vulnerability analyses, more work to understand farm scale diversity and other drivers of the differential between actual and expected yield would also be useful.

## Supporting Information

S1 Dataset Capital indicator, wheat yield and adaptive capacity index data. (XLSX)

S1 Supporting Information Results of the gladder procedure. Transformations with the lowest chi square value are best.
(DOCX)

S1 Table Results from the initial linear regression using all capital variables. (DOCX)

S2 Table Results from the fixed effects regression and t-tests using all capital variables. (DOCX)

S3 Table Results from the heteroscedasticity-robust nonlinear fixed effects regression and t-tests using all transformed capital variables. (DOCX)

S4 Table Results from the initial variance inflation factor test of all transformed variables. (DOCX)

S5 Table Results from the final variance inflation factor test of the refined set of transformed variables. (DOCX)
