# Supplementary material for: What Actually Confers Adaptive Capacity? Insights from Agro-Climatic Vulnerability of Australian Wheat
Source: PLoS One. 2015 Feb 10;10(2):e0117600. doi: 10.1371/journal.pone.0117600 (PMC4323342; doi:10.1371/journal.pone.0117600)

**Supporting Information S1. Results of the gladder procedure.** Transformations with the lowest chi square value are best.

Dependent variable – ACI, identity best (linear, no transformation required)


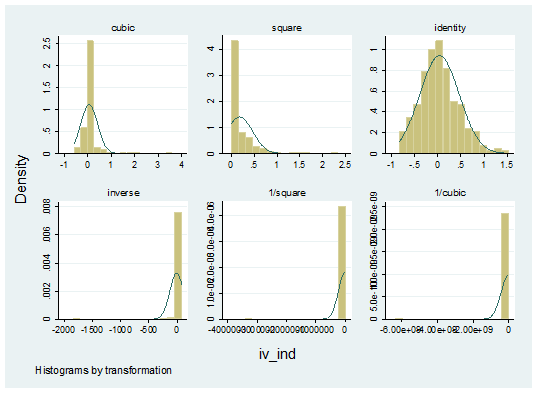
**
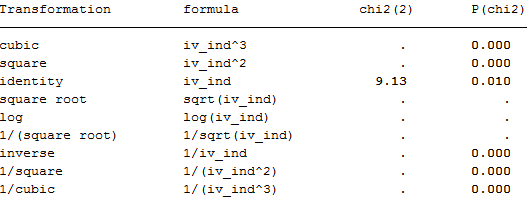
**

Explanatory variable – SC_FSFI, identity best (linear, no transformation required)


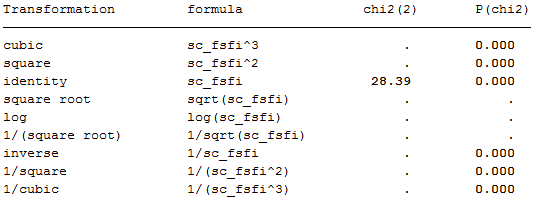


Explanatory variable – SC_Phone, inverse square root transformation best


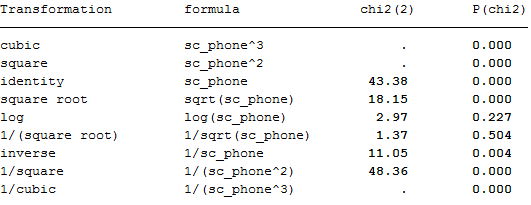


Explanatory variable – SC_FSFI, identity best (linear, no transformation required)


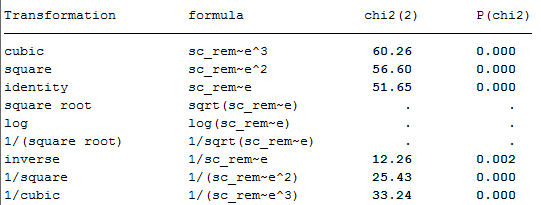


Explanatory variable – HC_Advis, square root transformation best


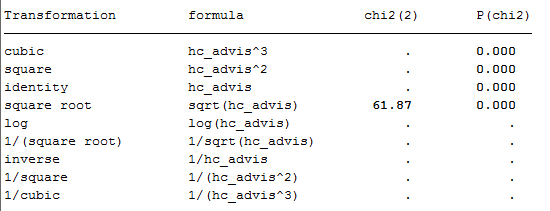


Explanatory variable – HC_CropDiv, identity best (linear, no transformation required)


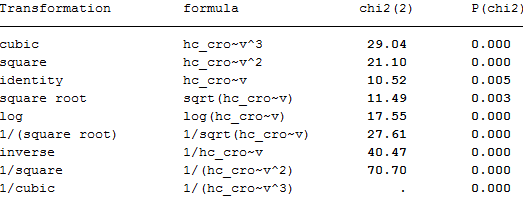


Explanatory variable – HC_LSDiv, log transformation best


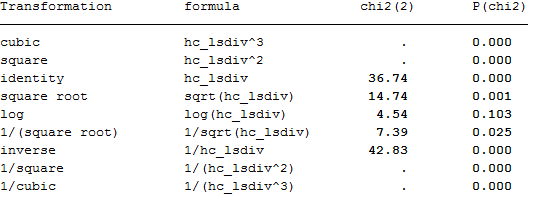


Explanatory variable – HC_Labor, log and square root only marginally better than identity. Identity selected due to easier interpretation


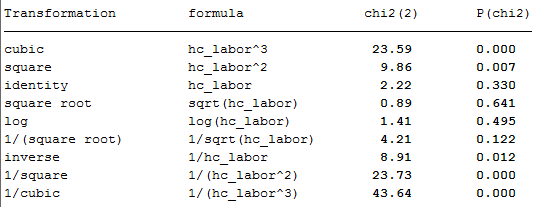


Explanatory variable – HC_Educ, cubic and square only marginally better than identity. Identity selected due to easier interpretation


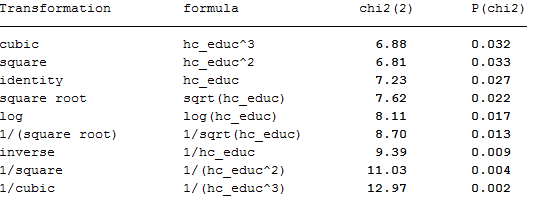


Explanatory variable – PC_VLFI, inverse square root only marginally better than log. Log selected due to easier interpretation


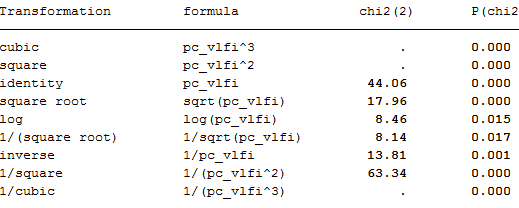


Explanatory variable – PC_Elec, square root only marginally better than identity. Identity selected due to easier interpretation


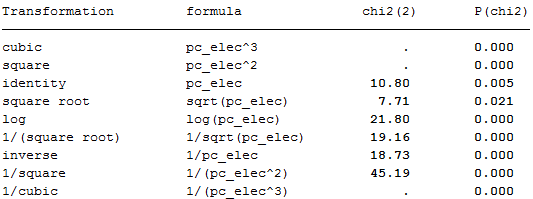


Explanatory variable – PC_Fert, log transformation best


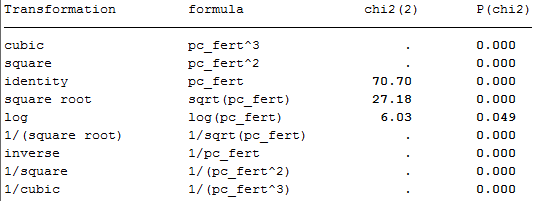


Explanatory variable – PC_Chem, log transformation best


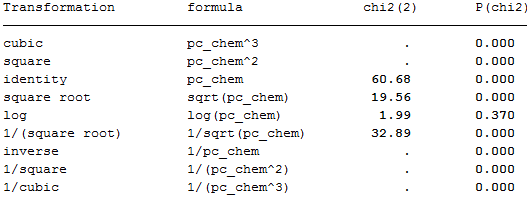


Explanatory variable – PC_Fuel, log transformation best


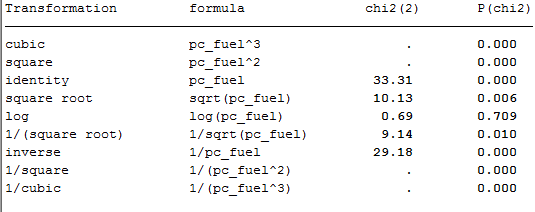


Explanatory variable – PC_Land, log transformation best


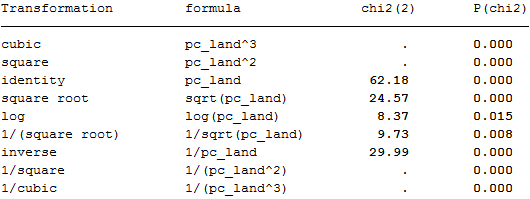


Explanatory variable – NC_MaxT, inverse cubic only marginally better than inverse square. Inverse square transformation selected due to easier interpretation


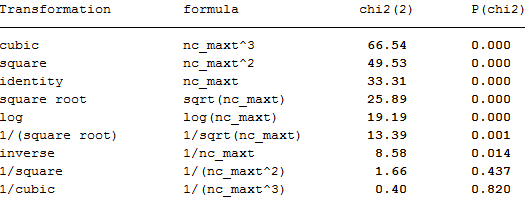


Explanatory variable – NC_SHRain, square root better than identity but identity still OK. Identity selected due to easier interpretation


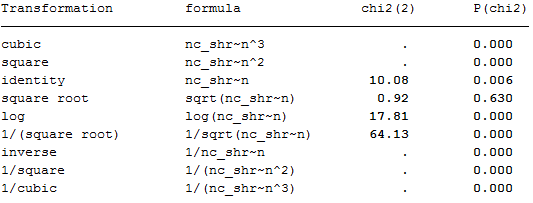


Explanatory variable – NC_TRain, square root and log better than identity but identity still OK. Identity selected due to easier interpretation


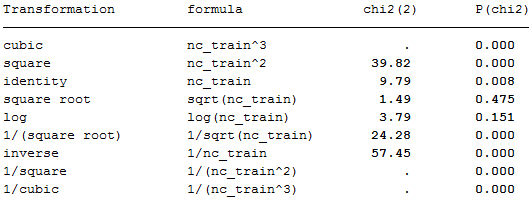


Explanatory variable – NC_SRad, inverse square root, log, and inverse only marginally better than identity. Identity selected due to easier interpretation


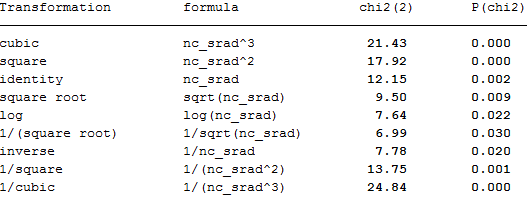


Explanatory variable – NC_SWHC, square only marginally better than identity. Identity selected due to easier interpretation


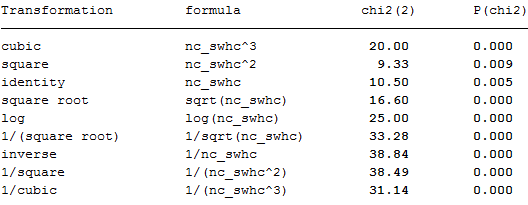


Explanatory variable – NC_NVeg, log transformation best


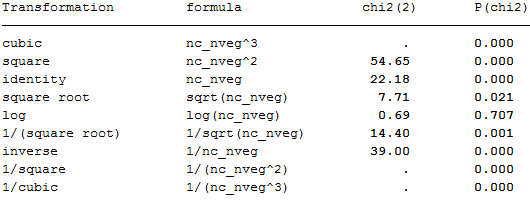


Explanatory variable – NC_NPP, inverse transformation best


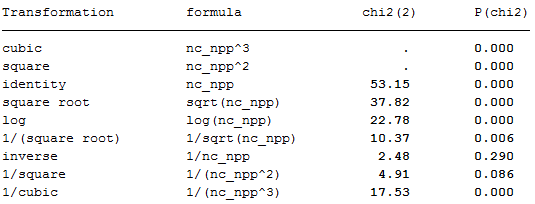


Explanatory variable – FC_TCCap, log transformation best


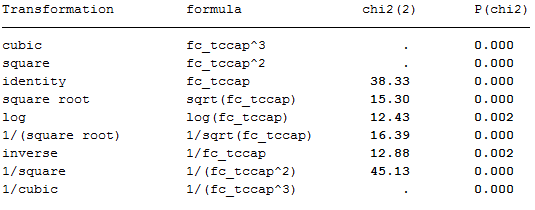


Explanatory variable – FC_AccCred, square only marginally better than log. Log selected due to easier interpretation


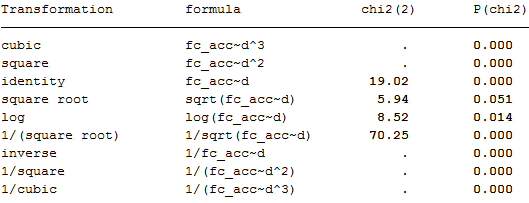


Explanatory variable – FC_RFInc, identity best (linear, no transformation required)


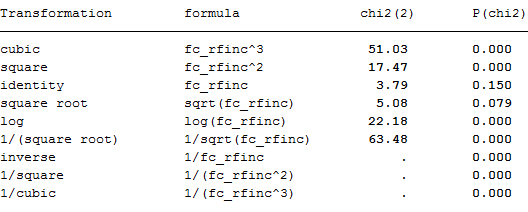

Supplement: S1 Supporting Information — Transformations with the lowest chi square value are best. (DOCX) [file pone.0117600.s002.docx]
