# Supplementary figures and images for: What Actually Confers Adaptive Capacity? Insights from Agro-Climatic Vulnerability of Australian Wheat
Source: PLoS One. 2015 Feb 10;10(2):e0117600. doi: 10.1371/journal.pone.0117600 (PMC4323342; doi:10.1371/journal.pone.0117600)

**Table S1. Results from the initial linear regression using all capital variables.**


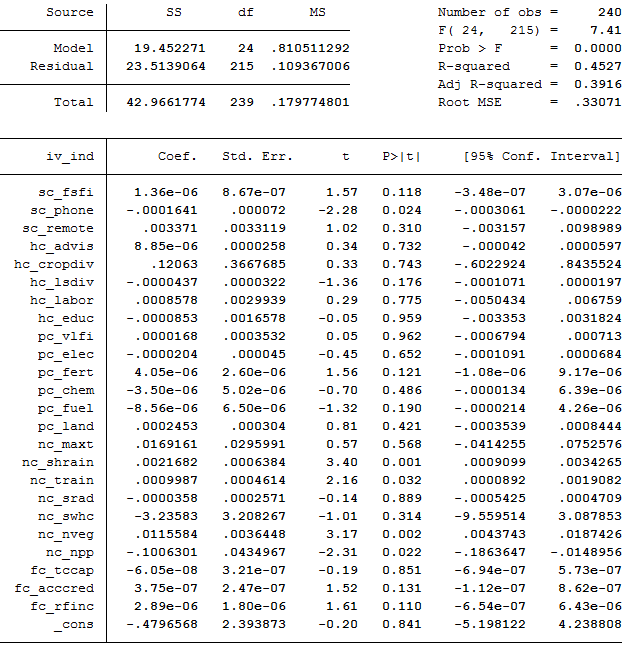

Supplement: S1 Table — (DOCX) [file pone.0117600.s003.docx]

**Table S2. Results from the fixed effects regression and *t*-tests using all capital variables.**


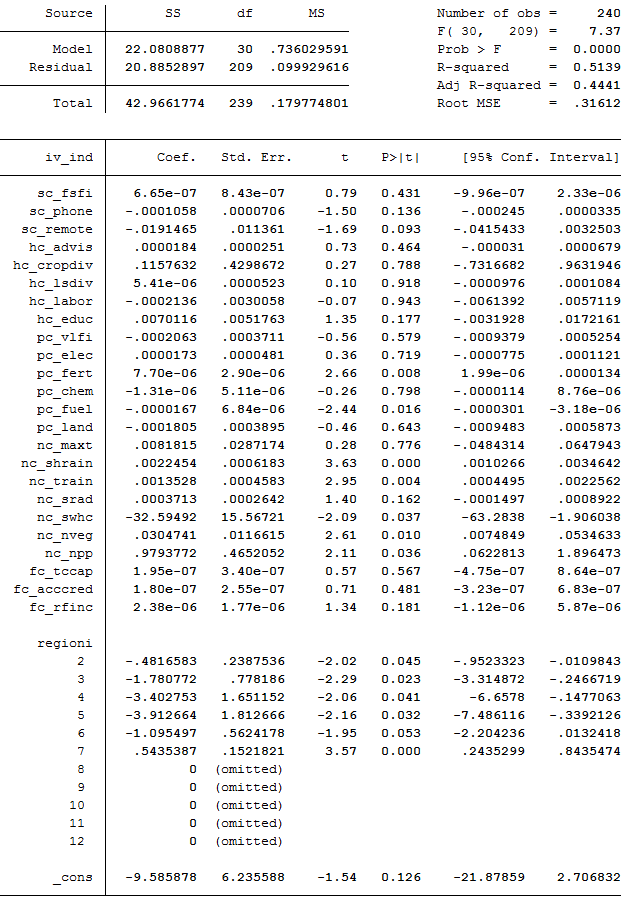

Supplement: S2 Table — (DOCX) [file pone.0117600.s004.docx]

**Table S4. Results from the initial variance inflation factor test of all transformed variables.**


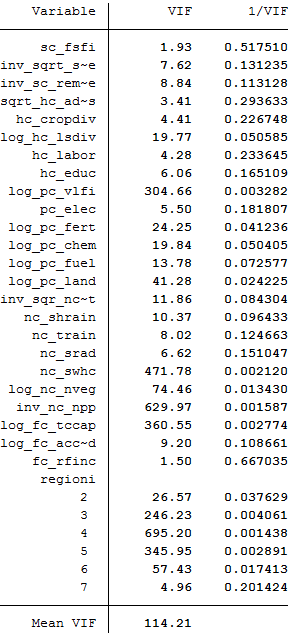

Supplement: S4 Table — (DOCX) [file pone.0117600.s006.docx]
