# Supplementary material for: What Actually Confers Adaptive Capacity? Insights from Agro-Climatic Vulnerability of Australian Wheat
Source: PLoS One. 2015 Feb 10;10(2):e0117600. doi: 10.1371/journal.pone.0117600 (PMC4323342; doi:10.1371/journal.pone.0117600)

**Table S3. Results from the heteroscedasticity-robust nonlinear fixed effects regression and *t*-tests using all transformed capital variables.**


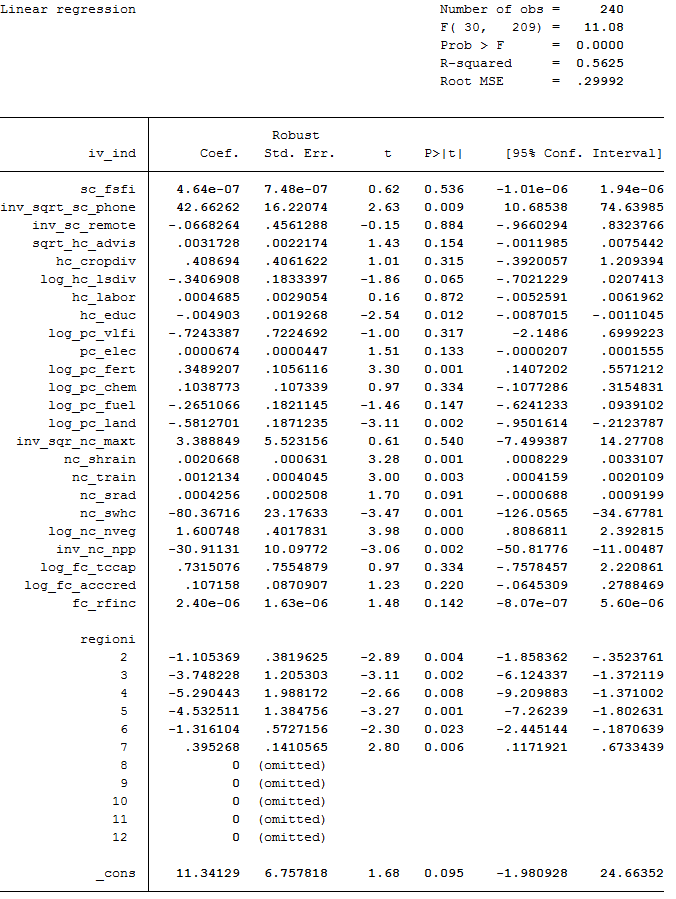

Supplement: S3 Table — (DOCX) [file pone.0117600.s005.docx]
