# Supplementary material for: What Actually Confers Adaptive Capacity? Insights from Agro-Climatic Vulnerability of Australian Wheat
Source: PLoS One. 2015 Feb 10;10(2):e0117600. doi: 10.1371/journal.pone.0117600 (PMC4323342; doi:10.1371/journal.pone.0117600)

**Table S5. Results from the final variance inflation factor test of the refined set of transformed variables.**


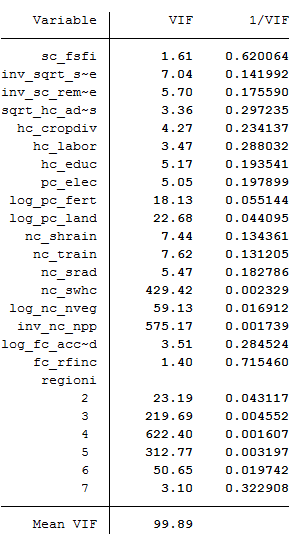

Supplement: S5 Table — (DOCX) [file pone.0117600.s007.docx]
